# Poussée de maladie de Kaposi et élévation du CA 19-9: penser à la tuberculose!

**DOI:** 10.11604/pamj.2013.16.81.2787

**Published:** 2013-11-05

**Authors:** Faida Ajili, Héla Hariz, Asmahen Souissi, Rim Abid, Najeh Boussetta, Besma Laabidi, Riadh Battikh, Bassem Louzir, Salah Othmani

**Affiliations:** 1 Service de Médecine Interne. Hôpital Militaire de Tunis, Tunisie; 2 Service d'anatomopathologie. Hôpital Militaire de Tunis, Tunisie

**Keywords:** Maladie de kaposi, tuberculose, CA 19-9, Kaposi disease, tuberculosis, CA 19-9

## Abstract

La maladie de Kaposi (MK) est une entité pathologique qui peut survenir chez les patients VIH positifs et dans le cadre d'une immunodépression, d'origine tuberculeuse très rarement. On décrit le cas d'une MK chez un patient VIH négatif au décours d'une tuberculose. Nous rapportons le cas d'un patient âgé de 81 ans, VIH négatif, ayant présenté deux nodules angiomateux de l'avant bras gauche dont la biopsie cutanée était en faveur d'une MK. L’évolution était marquée 2 mois plus tard, par l'apparition de placards angiomateux extensifs des deux membres supérieurs et d'adénopathies cervicales jugulo-carotidiennes bilatérales. La biopsie ganglionnaire était en faveur d'une tuberculose ganglionnaire. Par ailleurs, il avait un taux sérique élevé des CA 19-9. La régression de l’étendue des lésions au niveau des membres supérieurs et la normalisation du taux sérique des CA 19-9 ont été obtenues sous traitement anti-tuberculeux. Chez les patients atteints d'une MK avec une élévation des CA 19-9, il faut penser à la tuberculose.

## Introduction

La maladie de Kaposi (MK) est une entité pathologique qui revêt quatre formes cliniques: la forme classique méditerranéenne, la forme africaine, la forme associée au SIDA et la MK associée aux immunodépressions [1]. La pathogénie de la maladie de Kaposi fait intervenir de nombreux facteurs: la génétique, l´infection à HHV8 ainsi que les états d´immunodépression secondaires à l´infection au VIH, aux traitements immunosuppresseurs, aux syndromes lymphoprolifératifs et beaucoup plus rarement à la tuberculose [1, 2]. Nous rapportons ici le cas d'un patient âgé de 81 ans, VIH négatif, présentant une tuberculose ganglionnaire associée à une poussée une MK et à une élévation du taux sérique des CA 19-9.

## Patient et observation

**Figure 1:**
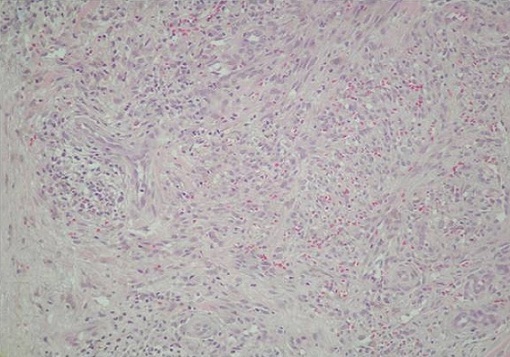
La biopsie cutanée du derme montrant une prolifération cellulaire fusiforme et vasculaire avec extravasation sanguine, associée à des éléments inflammatoires et de rares sidérophages avec un marquage nucléaire positif utilisant l'anticorps anti HHV8 concluant à une maladie de Kaposi.

**Figure 2:**
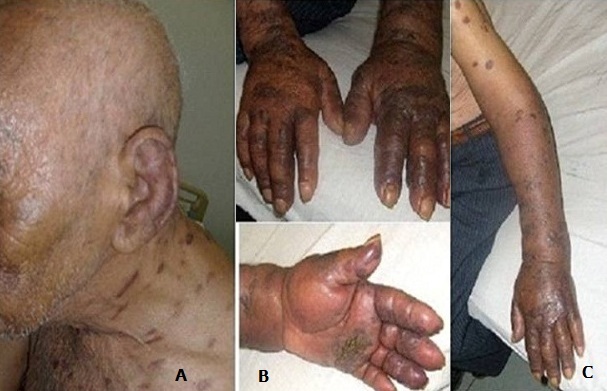
A: Lésions cutanées de la maladie de Kaposi au niveau du cou et du lobule de l'oreille. B: Localisation au niveau des 2 mains des lésions cutanées de la maladie de Kaposi réalisant a ce niveau des plaques extensives avec un lymphœdème des deux mains et lésions papillomateuses au niveau de la paume gauche. C: Lésions cutanées de la maladie de Kaposi au niveau d du membre supérieur gauche avec un lymphœdème de tout le membre.

**Figure 3:**
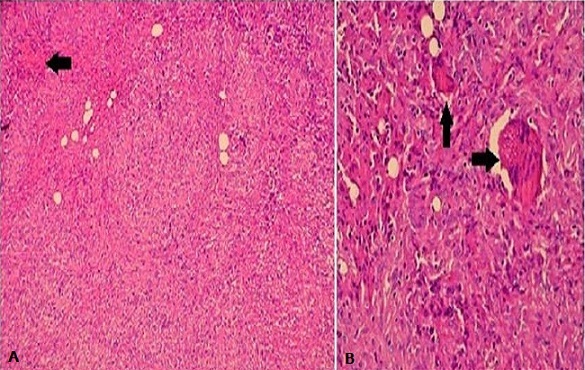
Biopsie ganglionnaire d'une adénopathie cervicale. 3A: (Coloration HES; Grossissement x10). La biopsie ganglionnaire montre une réaction inflammatoire chronique organisée en granulomes centrés, par endroit, par une nécrose caséeuse (flèche). 3B: (Coloration HES; Grossissement x40). Le granulome tuberculoïde comporte des cellules géantes de type Langhans (flèches) entourées de cellules épithéloides, de lymphocytes et de fibroblastes.

Mr SS, âgé de 81 ans, sans antécédents particuliers, présentait en Mai 2012 deux nodules angiomateux de 2 cm de diamètre au niveau de la face antérieure de l'avant bras gauche avec un lymphoedème de tout le membre. La biopsie cutanée de ces lésions avait montré au niveau du derme une prolifération cellulaire fusiforme et vasculaire avec extravasation sanguine, associée à des éléments inflammatoires et de rares sidérophages (Figure 1). L’étude immunohistochimique utilisant l'anticorps anti HHV8 montrait un marquage nucléaire positif des cellules endothéliales et des cellules fusiformes concluant ainsi à une maladie de Kaposi. La sérologie VIH était négative. L’évolution était marquée deux mois plus tard, par l'apparition de placards angiomateux au niveau de l'avant bras droit, du tronc et des lobules des 2 oreilles (Figure 2-A) avec une extension rapide au niveau de l'avant bras gauche et des 2 mains (Figure 2-B) associés à un lymphoedème important des 2 membres supérieurs prédominant à gauche (Figure 2-C +) et à des lésions papillomateuses au niveau de la paume de la main gauche (Figure 2-B). De façon concomitante, le patient avait présenté une altération progressive de l’état général, une fièvre et des sueurs nocturnes. L'examen clinique notait, outre les lésions cutanées sus décrites, des adénopathies cervicales jugulo-carotidiennes bilatérales de 1 à 2 cm de grand axe, fermes et fixes par rapport aux plans profonds. Il avait une anémie normochrome normocytaire arégénérative à 7.8 g/dl, des globules blancs à 4100 Elts/mm3 et une lymphopénie à 700 Elts/mm3. La vitesse de sédimentation était à 35mm à la première heure, la protéine C réactive était négative et la Ferritinémie était à 900µg /l (soit à 4 fois la normale). A l’électrophorèse des protéines plasmatiques on notait une hypoalbuminémie à 33g/l une hypergammaglobulinémie polyclonale à 19.7g/l. Les marqueurs tomoraux (ACE, α foetoprotéine, NSE) étaient normaux et le taux sérique des CA 19-9 à 163 UI/ml (valeur normale < 30 UI/ml). L'intradermo-réaction à la tuberculine, la recherche du Bacille de Koch dans les crachats et les urines étaient négatifs. Le scanner thoraco-abdomino-pelvien avait montré des adénopathies jugulocarotidiennes bilatérales nécrosées, un parenchyme pulmonaire sans anomalie et l'absence d'anomalies à l’étage abdominal. La fibroscopie digestive et la colonoscopie, réalisées devant le taux élevé des CA 19 9 étaient sans anomalies. La biopsie ganglionnaire concluait à une tuberculose ganglionnaire caséo-folliculaire (Figure 3). Le patient a été mis sous quadrithérapie anti-tuberculeuse, pendant 2 mois, à base d'Isoniazide (300 mg/j), Rifampicine (600 mg/j), Pyrazinamide (1.5 g/j) et Ethambutol (1 g/j) suivie d'une bithérapie anti-tuberculeuse (Isoniazide et Rifampicine). Quatre mois après, l’évolution était favorable sur le plan général, on notait une régression des adénopathies cervicales ainsi qu'une diminution de l’étendue des lésions cutanées et une régression partielle du lymphoedème au niveau des 2 membres supérieurs. Le taux des lymphocytes est passé de 700 à 1200 Elts/mm^3^ et le taux d'hémoglobine de 7.8 à 10.7g/dl, la ferritinémie s'est normalisée et le taux sérique des CA 19-9 est passé à 41.4UI/ml puis à 24.9UI/ml respectivement à J30 et J60 de traitement anti tuberculeux.

## Discussion

L'association de la MK à une TBC sur un terrain d'immunodépression a été rarement rapportée dans la littérature. Notre patient âgé de 81 ans, VIH négatif, a présenté une tuberculose ganglionnaire associée à une poussée une MK et à une élévation du taux sérique des CA 19-9.

Lanjewar et al [1] ont décrit le cas d'un patient âgé de 40 ans, VIH positif, ayant présenté une tuberculose multifocale (pulmonaire, hépatique, splénique) associée à une MK, diagnostiquées sur une même lame de biopsie ganglionnaire. Wang [2] a décrit le cas d'un patient qui a présenté 5 mois après une transplantation rénale, une tuberculose ganglionnaire associée à une maladie de kaposi extensive touchant la muqueuse buccale, le médiastin, le tube digestif et les poumons. Il a été mis sous traitement anti-tuberculeux et au bout d'une année, l’évolution était marquée par une régression spectaculaire de toutes les lésions de la MK.

La particularité de notre observation réside dans le fait que la MK est survenue en l'absence de tout autre facteur d'immunodépression que la tuberculose. Ceci a été très rarement rapporté dans la littérature, chez des patients ayant des formes florides de tuberculose pulmonaire [3], cutanée [4], voire des cas de miliaires induisant une immunodépression par un déficit de l'immunité cellulaire. D'ailleurs, l'amélioration des lésions kaposiennes sous traitement antituberculeux sans recours à un traitement spécifique conforte cette hypothèse. L'association d'une tuberculose ganglionnaire à une élévation du taux sérique des CA 19-9 constitue le second point original de notre observation. En effet, le CA 19.9 est reconnu comme étant le marqueur sérologique le plus spécifique de l'adénocarcinome pancréatique [5]. Les taux sériques du CA 19.9 peuvent cependant s’élever au cours de certaines affections non néoplasiques telles que les hépatites, les pancréatites, les syndromes cholestatiques quelle que soit leur origine [6] ainsi qu'au cours de certaines affections pulmonaires telles que la mucoviscidose [5] et les bronchectasies [7]. Dans de rares cas, la tuberculose est rapportée comme une étiologie de l’élévation non néoplasique du taux sérique des CA 19-9. Ceci a été observé chez des patients ayant une tuberculose pulmonaire ou péritonéale [8, 9, 10].

## Conclusion

Devant une malaldie de Kaposi extensive, la recherche d'un facteur d'immunodepression s'impose. Bien que rarement rapportée, la tuberculose doit être recherchée dans notre pays où cette affection est endémique. Par ailleurs, le marqueur tumoral CA 19-9 peut s’élever au cours d'une maladie tuberculeuse.
